# Anti-Inflammatory Effects of Adrenomedullin on Acute Lung Injury Induced by Carrageenan in Mice

**DOI:** 10.1155/2012/717851

**Published:** 2012-05-20

**Authors:** Talero Elena, Di Paola Rosanna, Mazzon Emanuela, Emanuela Esposito, Motilva Virginia, Cuzzocrea Salvatore

**Affiliations:** ^1^Department of Pharmacology, School of Pharmacy, University of Seville, 41012 Seville, Spain; ^2^Department of Clinical and Experimental Medicine and Pharmacology, School of Medicine, University of Messina, Torre Biologica, Policlinico Universitario, Via C. Valeria, Gazzi, 98100 Messina, Italy; ^3^IRCCS Centro Neurolesi “Bonino-Pulejo”, S.S. 113 Via Palermo, CTR Casazza, 98100 Messina, Italy; ^4^University of Manchester, Manchester M13 9PL, UK

## Abstract

Adrenomedullin (AM) is a 52 amino acid peptide that has shown predominant anti-inflammatory activities. In the present study, we evaluated the possible therapeutic effect of this peptide in an experimental model of acute inflammation, the carrageenan- (CAR-) induced pleurisy. Pleurisy was induced by injection of CAR into the pleural cavity of mice. AM (200 ng/kg) was administered by intraperitoneal route 1 h after CAR, and the animals were sacrificed 4 h after that. AM treatment attenuated the recruitment of leucocytes in the lung tissue and the generation and/or the expression of the proinflammatory cytokines as well as the expression of the intercellular cell adhesion molecules. Moreover, AM inhibited the induction of inducible nitric oxide synthase (iNOS), thereby abating the generation of nitric oxide (NO) and prevented the oxidative and nitroxidative lung tissue injury, as shown by the reduction of nitrotyrosine, malondialdehyde (MDA), and poly (ADP-ribose) polymerase (PARP) levels. Finally, we demonstrated that these anti-inflammatory effects of AM were associated with the inhibition of nuclear factor-**κ**B (NF-**κ**B) activation. All these parameters were markedly increased by intrapleural CAR in the absence of any treatment. We report that treatment with AM significantly reduces the development of acute lung injury by downregulating a broad spectrum of inflammatory factors.

## 1. Introduction

Injection of carrageenan (CAR) into the pleural space leads to local inflammation, infiltration by polymorphonuclear leukocytes (PMN), and lung injury [1]. This experimental model of pleurisy has been widely used to investigate the pathophysiology of acute inflammation and also to evaluate the efficacy of drugs in inflammation. In particular, the initial phase of acute inflammation. The acute reaction elicited by intrapleural injection of CAR is characterized by a marked accumulation of pleural exudate, measured by protein concentration, and intense migration of polymorphonuclear leukocytes (PMNs) into the pleural cavity [1]. PMNs moving out of the circulation into the inflamed tissue have a key function in the breakdown and remodeling of injured tissue [2]. In addition, oxidative stress is one of the earliest and most important components of acute lung injury induced by CAR and it is characterized by high levels of reactive oxygen species (ROS) and reactive nitrogen species (RNS). These molecules may lead to cell death by oxidative damage to DNA and proteins, lipid peroxidation in the cell membranes, and neutrophil recruitment on the injury tissue [3]. Furthermore, in the lungs, many noxious/inflammatory stimuli have been shown to activate transcription of nuclear factor *κ*B (NF-*κ*B), implicating this pathway as a focal point for induction of lung inflammation [4]. In normal conditions, NF-*κ*B, which is mainly composed of the subunits p50 and p65, is found in the cytoplasm of cells as inactive form bound to its inhibitor I*κ*B. After an inflammatory stimulus, I*κ*B kinases (IKK) phosphorylates I*κ*B, which will be degraded releasing NF-*κ*B that will be translocated to the nucleus to induce transcription of genes involved in the early onset of the inflammatory response [5].

Adrenomedullin (AM) is a 52-amino acid polypeptide initially isolated from human pheochromocytoma by Kitamura et al. [6]. AM is synthesized and secreted from a variety of tissues and organs, including adrenal medulla, lung, kidney, spleen, and heart. Accumulating evidence reveals the multifunctional biological activities of this peptide, which include vasorelaxation, bronchodilation, diuretic action, inhibition of aldosterone secretion, neurotransmission, antimicrobial activity, or growth regulation [7]. This peptide is found in low picomolar concentrations in the circulation and is reported to enhance in a coordinated fashion in experimental and clinical conditions of inflammation and sepsis [8].

Moreover, AM expression has shown to be increased in animals and humans with acute lung injury [9–11]. In the lungs, AM is expressed in many cell types, including bronchial epithelium, bronchial smooth muscles, pulmonary vasculature, and macrophages [12]. Plasma AM level is increased in a number of diseases, including essential hypertension, acute myocardial infarction, congestive heart failure, and renal failure [13, 14].

Recently Ceyhan et al. have measured the plasma levels of AM in asthma and in chronic obstructive airway disease and found that they were raised [15]. Plasma AM is markedly increased in endotoxic shock, suggesting that AM may be secreted in response to endotoxic shock [16] in sepsis [17], rheumatoid arthritis [18], and inflammatory bowel diseases [19]. Furthermore, anti-inflammatory effects of this peptide have been previously demonstrated in various models of lung disease. Along these lines, Itoh et al. [11] showed that AM infusion ameliorated LPS-induced acute lung injury in rats. In the same way, administration of AM after gut ischemia-reperfusion prevented acute lung injury through downregulation of proinflammatory cytokines [20]. Given that AM has emerged as a potential anti-inflammatory agent, the present study was designed to explore its possible therapeutic effect in an experimental model of CAR-induced pleurisy as well as to examine the underlying mechanisms responsible for its effects on this model of acute lung injury.

## 2. Material and Methods

### 2.1. Animals

Male CD mice (weight 20–25 g; Harlan Nossan, Milan, Italy) were used in these studies. The animals were housed in a controlled environment and provided with standard rodent chow and water. Animal care was in compliance with Italian regulations on the protection of animals used for experimental and other scientific purposes (D.M. 116192) as well as with EEC regulations (O.J. of E.C. L358/1 12/18/1986).

### 2.2. Carrageenan-Induced Pleurisy

Pleurisy by CAR was induced as previously described [21]. Mice were anaesthetized with isoflurane and subjected to a skin incision at the level of the left sixth intercostals space. The underlying muscle was dissected and saline (0.1 mL) or saline containing 2%  *λ*-CAR (0.1 mL) was injected into the pleural cavity. The skin incision was closed with a suture and the animals were allowed to recover. At 4 h after the injection of CAR, the animals were killed by inhalation of CO_2_. The chest was carefully opened and the pleural cavity rinsed with 1 mL of saline solution containing heparin (5 U mL^−1^) and indomethacin (10 *μ*g mL^−1^). The exudates and washing solution were removed by aspiration. Any exudates which were contaminated with blood were discarded.

### 2.3. Experimental Design

 Mice were randomly allocated into the following groups:

- *CAR + saline group*—mice were subjected to CAR-induced pleurisy (*N* = 10);
- *CAR + AM group*—same as the CAR + saline group but AM (200 ng/kg, i.p.) was administered 1 h after CAR (*N* = 10);
- *Sham + saline group*—sham-operated group in which identical surgical procedures to the CAR group was performed, except that the saline was administered instead of CAR (*N* = 10);
- *Sham + AM group*—same as the sham + saline group but AM (200 ng/kg, i.p.) was administered 1 h after saline solution (*N* = 10).

The dose of AM used (200 ng/kg, i.p.) was based on previous *in vivo* studies [19].

In a separate set of experiments, AM was administered in different doses in order to examine the dose-dependent therapeutic effect of adrenomedullin in a model of CAR-induced acute lung injury. Were used for this purpose the following groups:

- *CAR + saline group*—mice were subjected to CAR-induced pleurisy (*N* = 10)*; *
- *CAR + AM group*—same as the CAR + saline group but AM (100 ng/kg, i.p.) was administered 1 h after CAR (*N* = 10);
- *CAR + AM group*—same as the CAR + saline group but AM (50 ng/kg, i.p.) was administered 1 h after CAR (*N* = 10);
- *Sham + saline group*—sham-operated group in which identical surgical procedures to the CAR group was performed, except that the saline was administered instead of CAR (*N* = 10);
- *Sham + AM group*—same as the sham + saline group but AM (100 ng/kg, i.p.) was administered 1 h after saline solution (*N* = 10);
- *Sham + AM group*—same as the sham + saline group but AM (50 ng/kg, i.p.) was administered 1 h after saline solution (*N* = 10).

### 2.4. Histological Examination

Lung tissues samples were taken 4 h after injection of CAR. Lung tissue samples were fixed for 1 week in 10% (w/v) PBS-buffered formaldehyde solution at room temperature, dehydrated using graded ethanol and embedded in Paraplast (Sherwood Medical, Mahwah, NJ, USA). Sections were then deparaffinized with xylene, stained with hematoxylin and eosin. All sections were studied using Axiovision Zeiss (Milan, Italy) microscope. The following morphological criteria were used for scoring: 0, normal lung; grade 1, minimal oedema or infiltration of alveolar or bronchiolar walls; grade 3, moderate oedema and inflammatory cell infiltration without obvious damage to lung architecture; grade 4, severe inflammatory cell infiltration with obvious damage to lung architecture. All the histological studies were performed in a blinded fashion.

### 2.5. Measurement of Cytokines

IL-10, TNF-*α*, and IL-1*β* levels were evaluated in the exudates 4 h after the induction of pleurisy by CAR injection as previously described [22]. The assay was carried out using a colorimetric commercial ELISA kit (Calbiochem-Novabiochem Corporation, Milan, Italy). The ELISA has a lower detection limit of 5 pg/mL.

### 2.6. Measurement of Nitrite-Nitrate Concentration

Total nitrite in exudates, an indicator of NO synthesis, was measured as previously described [23]. Briefly, the nitrate in the sample was first reduced to nitrite by incubation with nitrate reductase (670 mU/mL) and *β*-nicotinamide adenine dinucleotide 3′-phosphate (NADPH) (160 *μ*M), at room temperature for 3 h. The total nitrite concentration in the samples was then measured using the Griess reaction, by adding 100 *μ*L of Griess reagent (0.1% w/v) naphthyl-ethylen-diamide-dihydrochloride in H_2_O and 1% (w/v) sulphanilamide in 5% (v/v) concentrated H_3_PO_4_; vol. 1 : 1) to the 100 *μ*l sample. The optical density at 550 nm (OD_550_) was measured using ELISA microplate reader (SLT-Lab Instruments, Salzburg, Austria). Nitrite concentrations were calculated by comparison with OD_550_ of standard solutions of sodium nitrite prepared in H_2_O and expressed as nmol/mL. 

### 2.7. Immunohistochemical Localization of Intercellular Cell Adhesion Molecule (ICAM-1) P-Selectin, iNOS, IL-1*β*, TNF-*α*, Nitrotyrosine, and Poly (ADP-Ribose) Polymerase (PARP)

 At the end of the experiment, the tissues were fixed in 10% (w/v) PBS-buffered formaldehyde and 8 *μ*m sections were prepared from paraffin-embedded tissues. After deparaffinization, endogenous peroxidase was quenched with 0.3% (v/v) hydrogen peroxide in 60% (v/v) methanol for 30 min. The sections were permeabilized with 0.1% (w/v) Triton X-100 in PBS for 20 min. Nonspecific adsorption was minimized by incubating the section in 2% (v/v) normal goat serum in PBS for 20 min. Endogenous biotin or avidin binding sites were blocked by sequential incubation for 15 min with both labels respectively. Sections were incubated overnight with anti-iNOS (1 : 500, Transduction Laboratories in PBS, v/v), anti-nitrotyrosine rabbit polyclonal antibody (Upstate, 1 : 500 in PBS, v/v), anti-PARP antibody (BioMol, 1 : 200 in PBS, v/v), anti-TNF-*α* ligand antibody (Santa Cruz Biotechnology, 1 : 500 in PBS, v/v), anti-IL-1*β* ligand antibody (Santa Cruz Biotechnology, 1 : 500 in PBS, v/v), anti-ICAM-1 antibody (Santa Cruz Biotechnology, 1 : 500 in PBS, v/v), or with anti-P-selectin polyclonal antibody (Santa Cruz Biotechnology, 1 : 500 in PBS, v/v). Sections were washed with PBS and incubated with secondary antibody. Specific labeling was detected with a biotin-conjugated goat anti-rabbit IgG and avidin-biotin peroxidase complex (Vector Laboratories, DBA). In order to confirm that the immunoreaction for the nitrotyrosine was specific, some sections were also incubated with the primary antibody (anti-nitrotyrosine) in the presence of excess nitrotyrosine (10 mM) to verify the binding specificity. To verify the binding specificity for iNOS, IL-1*β*, TNF-*α*, PARP, ICAM-1, P-selectin, some sections were also incubated with only the primary antibody (no secondary) or with only the secondary antibody (no primary). In these situations no positive staining was found in the sections indicating that the immunoreaction was positive in all the experiments carried out.

### 2.8. Myeloperoxidase (MPO) Activity

MPO activity, an indicator of PMN accumulation, was determined as previously described [24]. At the specified time following injection of CAR, lung tissues were obtained and weighed, each piece homogenized in a solution containing 0.5% (w/v) hexadecyltrimethyl-ammonium bromide dissolved in 10 mM potassium phosphate buffer (pH 7) and centrifuged for 30 min at 20,000 ×g at 4°C. An aliquot of the supernatant was then allowed to react with a solution of tetramethylbenzidine (1.6 mM) and 0.1 mM hydrogen peroxide. The rate of change in absorbance was measured spectrophotometrically at 650 nm. MPO activity was defined as the quantity of enzyme degrading 1 *μ*mol of peroxide min^−1^ at 37°C and was expressed in units per 100 mg of wet tissue.

### 2.9. Malondialdehyde (MDA) Measurement

MDA levels in the lung tissue were determined as an indicator of lipid peroxidation as previously described [25]. Lung tissue collected at the specified time, was homogenized in 1.15% (w/v) KCl solution. A 100 *μ*L aliquot of the homogenate was added to a reaction mixture containing 200 *μ*L of 8.1% (w/v) SDS, 1.5 mL of 20% (v/v) acetic acid (pH 3.5), 1.5 mL of 0.8% (w/v) thiobarbituric acid and 700 *μ*L distilled water. Samples were then boiled for 1 h at 95°C and centrifuged at 3,000 ×g for 10 min. The absorbance of the supernatant was measured using spectrophotometry at 650 nm. Results were expressed as *μ*M/100 mg wet tissue.

### 2.10. Western Blot Analysis for ICAM, P-Selectin, iNOS, I*κ*B-*α*, NF-*κ*B p65, IKK-*α*, PKA, and Phospho-NF-*κ*B (Ser536)

Cytosolic and nuclear extracts were prepared with slight modifications. Briefly, lung tissues from each mouse were suspended in extraction Buffer A containing 10 mM Hepes, 10 mM KCl, 0.1 mM EDTA, 0.1 mM EGTA, 1 mM DTT, 0.5 mM PMSF, 3 *μ*g/mL pepstatin A, 2 *μ*g/mL leupeptin, 15 *μ*g/mL Trypsin inhibitor, 40 *μ*M Benzamidine, homogenized at the highest setting for 2 min, and centrifuged at 13,000 ×g for 3 min at 4°C. Supernatants represented the cytosolic fraction. The pellets, containing enriched nuclei, were resuspended in Buffer B containing 20 mM Hepes, 1.5 mM MgCl_2_, 0.4 M NaCl, 1 mM EGTA, 1 mM EDTA, 1 mM DTT, 0.5 mM PMSF, 3 *μ*g/mL pepstatin A, 2 *μ*g/mL leupeptin, 15 *μ*g/mL Trypsin inhibitor, 40 *μ*M Benzamidine, 1% NONIDET P40, 20% Glycerol. After centrifugation 10 min a 13,000 ×g at 4°C, the supernatants containing the nuclear protein were stored at −80°C for further analysis. The levels of ICAM, P-selectin, I*κ*B-*α*, iNOS, IKK-*α*, PKA, and phospho-NF-*κ*B (ser536) were quantified in cytosolic fraction from lung tissue collected 4 h after CAR administration, while NF-*κ*B p65 levels were quantified in nuclear fraction. Protein concentration in cell lysates was determined by Bio-Rad Protein Assay (BioRad, Richmond CA, USA) and 50 *μ*g of cytosol and nuclear extract from each sample was analyzed. Proteins were separated by a 12% SDS-polyacrylamide gel electrophoresis and transferred on PVDF membrane (Hybond-P, Amershan Biosciences, UK). The membrane was blocked with 0.1% TBS-Tween containing 5% nonfat milk for 1 h at room temperature and subsequently probed with specific Abs ICAM (Santa Cruz Biotechnology, 1 : 500), P-selectin (Santa Cruz Biotechnology, 1 : 500), I*κ*B-*α* (Santa Cruz Biotechnology, 1 : 1000), or anti-iNOS (1 : 1000; Transduction) or anti-NF-*κ*B p65 (1 : 1000; Santa Cruz Biotechnology) IKK-*α* (Santa Cruz Biotechnology, 1 : 1000), PKA (Santa Cruz Biotechnology, 1 : 1000) and phospho-NF-*κ*B (ser536) (Santa Cruz Biotechnology, 1 : 1000) in 1x PBS, 5% w/v nonfat dried milk, 0.1% Tween-20 (PMT) at 4°C, overnight. Membranes were incubated with peroxidase-conjugated bovine anti-mouse IgG secondary antibody or peroxidase-conjugated goat anti-rabbit IgG (1 : 2000, Jackson ImmunoResearch, West Grove, PA, USA) for 1 h at room temperature. To ascertain that blots were loaded with equal amounts of proteic lysates, they were also incubated in the presence of the antibody against *β*-actin protein (1 : 10,000 Sigma-Aldrich Corp.) and anti-lamin B1 (1 : 10,000 Sigma-Aldrich Corp.). Protein bands were detected with Super-Signal West Pico Chemiluminescent (PIERCE). The relative quantification of the protein bands of ICAM(~110 kDa), P-selectin(~140 kDa), I*κ*B-*α* (~37 kDa), NF-*κ*B p65 (~65 kDa), iNOS (~130 kDa), IKK-*α* (~85 kDa), PKA (~40 kDa), and phospho-NF-*κ*B (ser536) (~65 kDa) was quantified by densitometric scanning of the X-ray films with GS-700 Imaging Densitometer (GS-700, Bio-Rad Laboratories, Milan, Italy) and a computer program (Molecular Analyst, IBM), and standardized for densitometric analysis to *β*-actin and lamin B1 protein levels. 

### 2.11. Materials

Unless otherwise stated, all compounds were obtained from Sigma-Aldrich Company Ltd. (Poole, Dorset, UK). AM was obtained from Bachem (St. Helens, UK). All other chemicals were of the highest commercial grade available. All stock solutions were prepared in nonpyrogenic saline (0.9% NaCl; Baxter, Italy, UK).

### 2.12. Statistical Evaluation

All values in the figures and text are expressed as mean ± standard error (s.e.m.) of the mean of n observations. For the *in vivo* studies *n* represents the number of animals studied. In the experiments involving histology or immunohistochemistry, the figures shown are representative of at least three experiments (histological or immunohistochemistry coloration) performed on different experimental days on the tissue sections collected from all the animals in each group. The results were analyzed by one-way analysis of variance (ANOVA) followed by Bonferroni's post hoc test for multiple comparisons and assessed with two-way anova for repeated measures and followed by Student's *t*-test. A *P* value of less than 0.05 was considered statistically significant.

## 3. Results

### 3.1. Effects of AM on CAR-Induced Pleurisy

**Figure 1:**
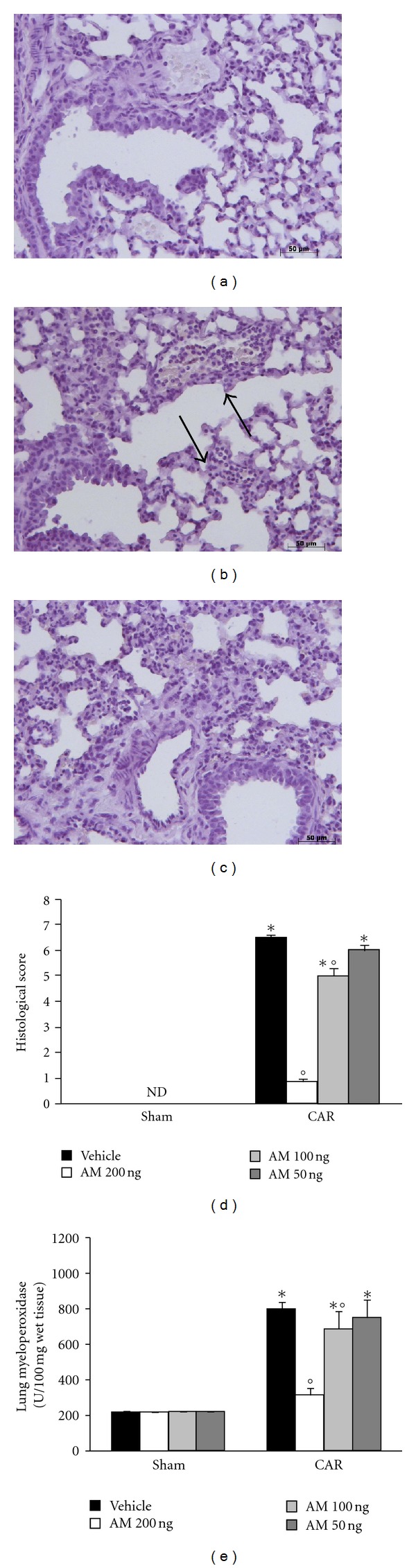
Effect of adrenomedullin (AM) on histological alterations and on PMN infiltration in the lung. Lung sections taken from CAR-treated mice treated with vehicle demonstrated edema, tissue injury (b, d), as well as infiltration of the tissue with neutrophils (b). CAR-treated animals treated with AM (c) demonstrated reduced lung injury and neutrophil infiltration in a dose-dependent manner. Section from sham animals showed the normal architecture of the lung tissue (a). The histological score (d) was made by an independent observer. MPO activity, index of PMN infiltration, was significantly elevated at 4 h after CAR administration in vehicle-treated mice (e), if compared with sham mice (e). AM significantly reduced MPO activity in the lung in a dose-dependent manner (e). The figure is representative of at least 3 experiments performed on different experimental days. Data are expressed as mean ± s.e.m. from *n* = 10 mice for each group. _  _*P < 0.01 versus sham group. _  _°*P* < 0.01 versus CAR.

When compared to lung sections taken from saline-treated animals (sham group, Figure 1(a), see densitometry analysis in Figure 1(d)), histological examination of lung sections taken from mice treated with CAR revealed significant tissue damage and edema (Figure 1(b), see histological score in Figure 1(d)), as well as infiltration of neutrophils (PMNs) within the tissues (Figure 1(b)). However, AM reduced in a dose-dependent manner the degree of lung injury (Figure 1(c), see histological score in Figure 1(d)).

The pleural infiltration with PMN appeared to correlate with an influx of leukocytes into the lung tissue, thus we investigated the effect of AM on neutrophil infiltration by measurement of MPO activity; This enzyme activity was significantly elevated at 4 h after CAR administration in vehicle-treated mice (Figure 1(e)). Treatment with AM significantly attenuated in a dose-dependent manner neutrophil infiltration into the lung tissue (Figure 1(e)).

### 3.2. Effects of AM on the Expression of Adhesion Molecules (ICAM-1, P-Selectin)

**Figure 2:**
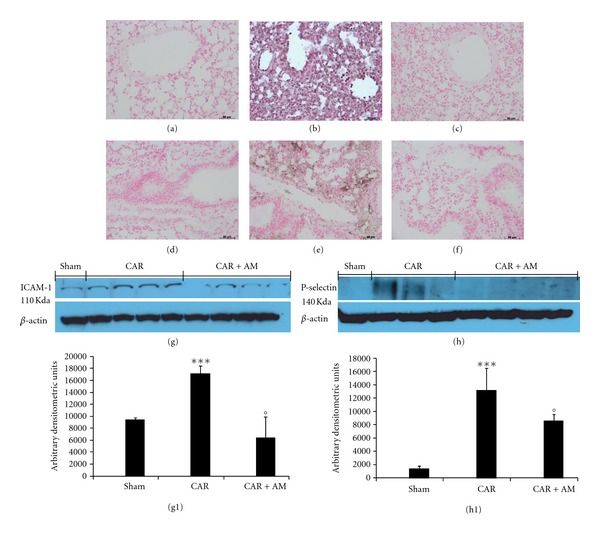
Effect of adrenomedullin (AM) on the immunohistochemical localization of ICAM-1 and P-selectin. No positive staining for ICAM-1 was observed in lung sections taken from sham mice treated with AM (a). Lung sections taken from CAR-treated mice showed intense positive staining for ICAM-1 along the vessels (b). The degree of positive staining for ICAM-1 was markedly reduced in lung sections obtained from mice treated with AM (c). No positive staining for P-selectin was observed in lung sections taken from sham mice (d). Lung sections taken from CAR-treated mice treated with vehicle showed intense positive staining for P-selectin along the vessels (e). The degree of positive staining for P-selectin was markedly reduced in tissue sections obtained from mice treated with AM (f). The figure is representative of at least three experiments performed on different experimental days. A significant increase in ICAM (g, g1) and P-selectin (h, h1) quantification, assayed by western blot analysis, was detected in lungs obtained from mice subjected to CAR-induced pleurisy, if compared with lung from sham mice (g, g1 and h, h1). Treatment with AM significantly attenuated these proteins quantification in the lung tissues (g, g1 and h, h1). A representative blot of lysates obtained from 5 animals per group is shown and densitometry analysis of all animals is reported. The results in panel (g1) and (h1) are expressed as mean ± s.e.m. from *n* = 5/6 lung tissues for each group. _  _*P < 0.01 versus sham group. _  _°*P* < 0.01 versus CAR.

No positive staining for ICAM-1 and P-selectin was found in lung tissue sections from saline-treated mice (Figures 2(a) and 2(d), resp.). At 4 h after CAR injection, the ICAM-1 staining intensity increased in the vascular endothelium (Figure 2(b)). In the same line, lung tissue sections obtained from CAR-treated mice showed positive staining for P-selectin localized in the vessels (Figure 2(e)). No positive staining for ICAM-1 or P-selectin was observed in the lungs of CAR-treated mice pretreated with AM (Figures 2(c) and 2(f), resp.). Western blotting study confirmed results from immunohistochemical analysis, showing a significant increase in ICAM-1 (Figure 2(g) see densitometry Figure 2(g1)) and P-selectin (Figure 2(h) see densitometry Figure 2(h1)) quantification in lungs taken from mice subjected to CAR-induced pleurisy. However, in AM-treated mice a lower quantification of these proteins could be detected (Figures 2(g) and 2(h) see densitometry Figures 2(g1) and 2(h1)).

### 3.3. Effects of AM on iNOS Expression and Nitrite-Nitrate Concentration

**Figure 3:**
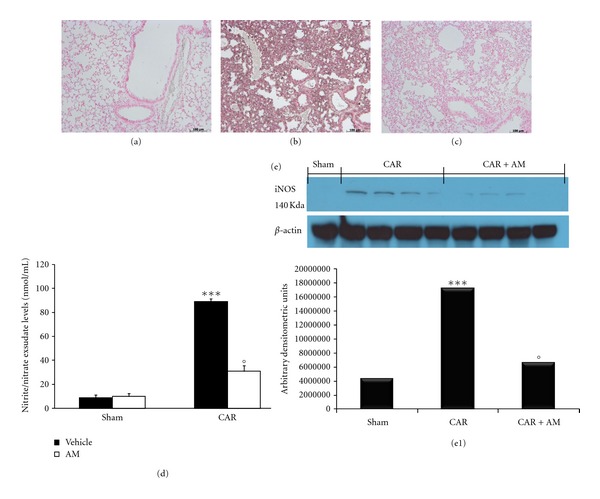
Effect of adrenomedullin (AM) on iNOS expression and NO formation. Lung sections taken from CAR-treated mice treated with vehicle showed positive staining for iNOS, localized mainly in inflammatory cells (b). The degree of positive staining for iNOS was markedly reduced in tissue sections obtained from mice treated with AM (c). Lung sections taken from sham mice showed no staining for iNOS (a). The figure is representative of at least 3 experiments performed on different experimental days. A significant increase in iNOS (e, e1) quantification, assayed by Western blot analysis, was detected in lungs obtained from mice subjected to CAR-induced pleurisy, if compared with lung from sham mice (e, e1). Treatment with AM significantly attenuated this protein quantification in the lung tissues (e, e1). A representative blot of lysates obtained from 5 animals per group is shown and densitometry analysis of all animals is reported. The results in panel (e1) are expressed as mean ± s.e.m. from *n* = 5/6 lung tissues for each group. *P < 0.01 versus sham group. °*P* < 0.01 versus CAR. Nitrite and nitrate levels, stable NO metabolites, were significantly increased in the pleural exudates at 4 h after CAR (CAR) administration (d). AM significantly reduced the CAR-induced elevation of nitrite and nitrate in the exudates (d). Data are expressed as mean ± s.e.m. from *n* = 10 mice for each group. _  _*P < 0.01 versus sham group. _  _°*P* < 0.01 versus CAR.

No positive staining for iNOS was observed in the lung tissues obtained from the sham group (Figure 3(a)). Immunohistochemical analysis of lung sections obtained from CAR-treated mice revealed positive staining for iNOS (Figure 3(b)). AM treatment (200 ng/kg) significantly attenuated this protein expression (Figure 3(c)). Western blotting study confirmed results from immunohistochemical analysis, showing a significant increase in iNOS quantification in lungs taken from mice subjected to CAR-induced pleurisy. However, in AM-treated mice a lower quantification of this inducible protein could be detected (Figure 3(e), see densitometry analysis in Figure 3(e1)). NO levels were also significantly increased in the exudates obtained from mice administered CAR in comparison with those from sham animals (Figure 3(d)). By contrast, treatment of mice with AM significantly reduced NO levels (Figure 3(d)).

### 3.4. Effects of AM on CAR-Induced Nitrotyrosine Formation, Lipid Peroxidation, and PARP Activation

**Figure 4:**
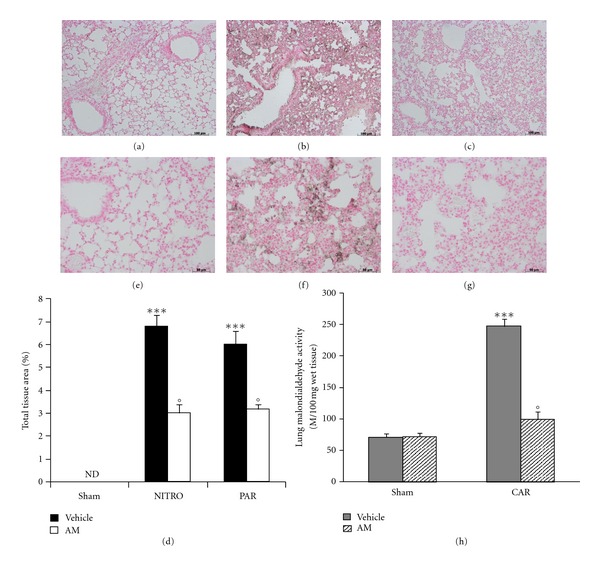
Effect of adrenomedullin (AM) on nitrotyrosine formation, lipid peroxidation, and PARP activation. No staining for nitrotyrosine is present in lung section from sham mice (a). Lung sections taken from CAR-treated mice treated with vehicle showed positive staining for nitrotyrosine, localized mainly in inflammatory cells (b). There was a marked reduction in the immunostaining for nitrotyrosine in the lungs of CAR-treated mice treated with AM (c). Malondialdehyde (MDA) levels, an index of lipid peroxidation, were significantly increased in lung tissues 4 h after CAR administration (h), if compared with lung from sham mice (h). AM significantly reduced the CAR-induced elevation of MDA tissue levels (h). Lung sections taken from CAR-treated mice showed positive staining for PAR (f). There was a marked reduction in the immunostaining for PAR in the lungs of CAR-treated mice treated with AM (g). Lung section from sham mice showed no staining for PAR (e). Densitometry analysis of immunocytochemistry photographs (*n* = 5 photos from each sample collected from all mice in each experimental group) for nitrotyrosine and PAR from lung tissues was assessed (d). The assay was carried out by using Optilab Graftek software on a Macintosh personal computer (CPU G3-266). Data are expressed as % of total tissue area. The figure is representative of at least 3 experiments performed on different experimental days. Data are expressed as mean ± s.e.m. from *n* = 10 mice for each group. _  _*P < 0.01 versus sham group. _  _°*P* < 0.01 versus CAR.

Immunohistochemical analysis of lung sections obtained from mice treated with CAR revealed positive staining for nitrotyrosine (Figure 4(b) see densitometry Figure 4(d)). In contrast, no positive staining for nitrotyrosine was found in the lungs of CAR-treated mice, which had been treated with AM (200 ng/kg) (Figure 4(c) see densitometry Figure 4(d)). At the same time point (4 h after CAR administration), lung tissue sections were taken in order to determine the immunohistological staining for poly ADP-ribosylated proteins (an indicator of PARP activation). A positive staining for the PARP (Figure 4(f) see densitometry Figure 4(d)) was found primarily localized in the inflammatory cells present in the lung tissue from CAR mice. AM reduced the degree of PARP activation (Figure 4(g) see densitometry Figure 4(d)). Note that there was no staining for either nitrotyrosine (Figure 4(a) see densitometry Figure 4(d)) or PAR (Figure 4(e) see densitometry Figure 4(d)) in lung tissues obtained from the sham group of mice. In addition, at 4 hours after the pleurisy was induced, MDA levels were also measured in the lungs as an indicator of lipid peroxidation. As shown in Figure 4(h), MDA levels were significantly increased in the lungs of CAR-treated mice. Lipid peroxidation was significantly attenuated by the intraperitoneal injection of AM (Figure 4(h)).

### 3.5. Effects of AM on the Release and Expression of Proinflammatory Cytokines Induced by CAR

**Figure 5:**
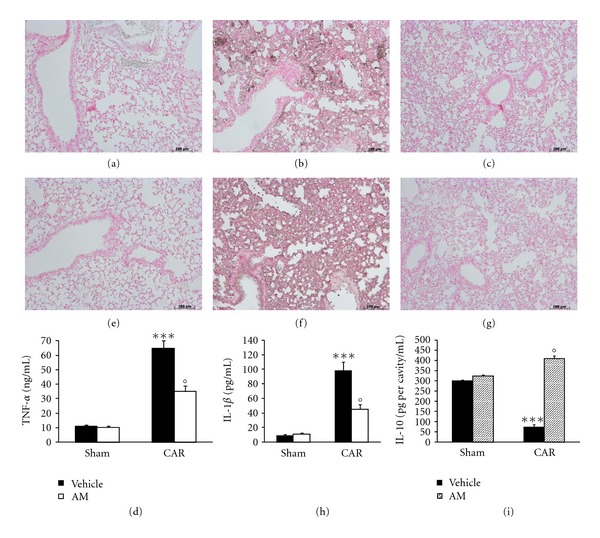
Effect of adrenomedullin (AM) on proinflammatory cytokine expression and release. Lung sections taken from CAR-treated mice showed positive staining for TNF-*α* and IL-1*β* (b and f, resp.). There was a marked reduction in the immunostaining for TNF-*α* and IL-1*β* in the lungs from CAR-treated mice treated with AM (c and g, resp.). No staining for either TNF-*α* (a) or IL-1*β* (e) in lung tissues obtained from the sham group was detected. The figure is representative of at least 3 experiments performed on different experimental days. Moreover, pleural injection of CAR caused an increase in exudate levels of TNF-*α* (d) and IL-1*β* (h). AM significantly reduced TNF-*α* and Il-1b levels (d and h, resp.). Finally IL-10 were measured in the lung exudates. In CAR-treated mice, however, significant decreases (*P* < 0.01) in the levels of IL-10, in comparison to values obtained inAM-treated mice, were detected (i). Data are means ± s.e.m. of 10 mice for each group. _  _*P < 0.01 versus sham group. _  _°*P* < 0.01 versus CAR.

When compared to sham animals, injection of CAR resulted in an increase in the levels of TNF-*α* and IL-1*β* in the pleural exudates (Figures 5(d) and 5(h), resp.). The release of these proinflammatory cytokines was significantly attenuated by treatment with AM (Figures 5(d) and 5(h), resp.). Therefore, we also evaluate the TNF-*α* and IL-1*β* expression in the lung tissues by immunohistochemical detection. Tissue sections obtained from vehicle-treated animals at 4 h after CAR injection demonstrate positive staining for TNF-*α* mainly localized in the infiltrated inflammatory cells, pneumocytes as well as in vascular wall (Figure 5(b)). In contrast, no staining for TNF-*α* was found in the lungs of CAR-treated mice that had been also treated with AM (Figure 5(c)). Similarly, positive staining for IL-1*β*, mainly, localized in the infiltrated inflammatory cells, was observed in lung tissue sections obtained from vehicle-treated animals (Figure 5(f)). AM treatment reduced the degree of IL-1*β* expression (Figure 5(g)). Please note that there was no staining for either TNF-*α* (Figure 5(a)) or IL-1*β* (Figure 5(e)) in lung tissues obtained from the sham group of mice. To further characterize the carrageenan-induced acute lung inflammation in AM-treated mice, the anti-inflammatory cytokine IL-10 were measured in the lung exudates. In CAR-treated mice however, significant decreases (*P* < 0.01) in the levels of IL-10, in comparison to values obtained in AM-treated mice, were detected (Figure 5(i)).

### 3.6. Effect of AM on I*κ*B-*α* Degradation NF-*κ*B p65 Activation and IKK-*α*, PKA, and Phospho-NF-*κ*B (Ser536)

**Figure 6:**
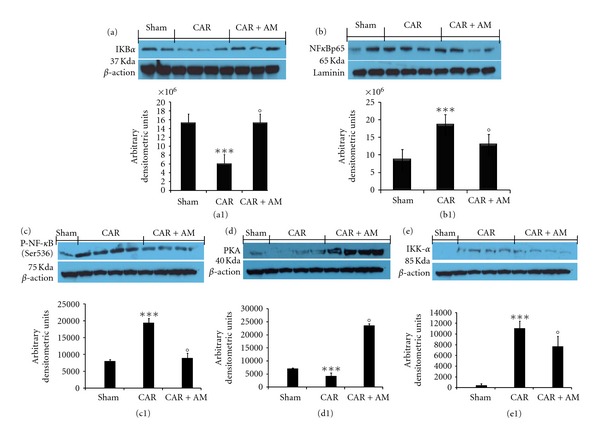
Effect of adrenomedullin (AM) on I*κ*B-*α* degradation, nuclear NF-*κ*B p65 quantification, IKK-*α*, PKA, and phospho-NF-*κ*B (ser536). Basal level of I*κ*B-*α* was detected in lung samples from sham-treated animals, whereas I*κ*B-*α* levels were substantially reduced in lung tissues obtained from vehicle-treated animals at 4 h after CAR injection (a, a1). AM treatment prevented CAR-induced I*κ*B-*α* degradation (a, a1). NF-*κ*B p65 levels in the lung nuclear fractions were significantly increased at 4 h after CAR injection compared to the sham-treated mice (b, b1). AM treatment significantly reduced the levels of NF-*κ*B p65 (b, b1). AM significantly reduced the levels of IKK alpha (e, e1) and the phosphorylation of p65 on Ser536 (c, c1). Whereas prevented CAR-induced PKA decreases (d, d1). A representative blot of lysates obtained from 5 animals per group is shown and densitometry analysis of all animals is reported. The results in panel (a1, b1, c1, d1 and e1) are expressed as mean ± s.e.m. from *n* = 5/6 lung tissues for each group. _  _*P < 0.01 versus sham group. _  _°*P* < 0.01 versus CAR.

We evaluated the quantification of I*κ*B-*α* and nuclear NF-*κ*B p65 by western blot analysis to investigate the cellular mechanisms whereby treatment with AM attenuates the development of acute lung injury. Basal levels of I*κ*B-*α* was detected in lung samples from sham-treated animals, whereas I*κ*B-*α* levels were substantially reduced in lung tissues obtained from vehicle-treated animals at 4 h after CAR injection (Figure 6(a), see densitometry analysis Figure 6(a1)). AM (200 ng/kg) treatment prevented CAR-induced I*κ*B-*α* degradation (Figure 6(a), see densitometry analysis in Figure 6(a1)). Moreover, NF-*κ*B p65 levels in the lung nuclear fractions were also significantly increased at 4 h after CAR injection compared to the sham-treated mice (Figure 6(b), see densitometry analysis in Figure 6(b1)). AM significantly reduced the levels of NF-*κ*B p65, as shown in Figure 6(b) (see densitometry analysis in Figure 6(b1)). To confirm the nuclear translocation of active NFkB we also evaluated the quantification of active IKK complex, in particular of IKK alpha and phospho-NF-*κ*B (ser536). As shown in Figure 6(e), AM significantly reduced the levels of IKK-*α* (see densitometry analysis in Figure 6(e1)). A significant increase in the phosphorylation of Ser536 was observed in CAR-treated mice (Figure 6(c), see densitometry analysis Figure 6(c1)). The treatment with AM resulted in a significant decrease of the phosphorylation of p65 on Ser536 (Figure 6(c), see densitometry analysis Figure 6(c1)). In addition to explaining the AM interactions, we evaluated the quantification of activates protein kinase A (PKA). Whereas PKA levels were substantially reduced in lung tissues obtained from vehicle-treated animals at 4 h after CAR injection (Figure 6(d), see densitometry analysis Figure 6(d1)). AM (200 ng/kg) treatment prevented CAR-induced PKA decreases (Figure 6(d), see densitometry analysis in Figure 6(d1)).

## 4. Discussion

Neuropeptides produced during an ongoing inflammatory response have emerged as endogenous anti-inflammatory agents that participate in processes leading to the resolution of inflammation and maintenance of tolerance. In this line, AM is a vasorelaxant neuropeptide that has shown to be an endogenous immunomodulatory factor, with predominant anti-inflammatory activities in different experimental conditions. These findings suggest that this peptide could be an interesting potential alternative for the treatment of immunological disorders [26, 27]. Considering these beneficial effects of AM, the present study investigated the extent to which the administration of the peptide would protect the lung in a model of CAR-induced acute lung injury in mice. The intrapleural injection of the polysaccharide CAR elicits an inflammatory response characterized by accumulation of fluid and migration of leukocytes to the site of the inflammation [1]. Analysis of our results revealed the anti-inflammatory actions of this peptide in the experimental model of pleurisy. Edema and lung tissue damage were attenuated when AM was administered i.p. at the dose of 200 ng/kg. Moreover, this peptide caused significant inhibitory effects on neutrophil influx into the pleural cavity 4 hour after CAR administration, as assessed by the histological analysis and in the specific granulocyte enzyme MPO activity study. These results are in line with previous papers which demonstrated that AM exerts a significant protective effect in lung injury. In this way, Itoh et al. [11] showed that infusion of AM ameliorated LPS-induced acute lung injury in rats. Moreover, a recent study reported the beneficial effect of the peptide on ventilator-induced lung injury in mice, due in part to the attenuation of the accumulation of leucocytes in the lung [28]. The maintenance of leukocyte recruitment during inflammation requires intercellular communication between infiltrating leukocytes and the endothelium. These events are mediated by the generation of early response cytokines, for example, IL-1*β* and TNF-*α*, which act on the endothelial cell receptors inducing NO and cytokines production and cellular adhesion molecules expression in neutrophils [29]. We have already demonstrated in previous study that TNF-*α* and IL-1*β* appear to play a critical role in the induction of inflammatory responses by initiating cytokine and chemokine cascades [30–32]. Blocking of either of these cytokines results in profound reductions in neutrophil influx and in the intensity of lung injury [33]. In accordance with these findings, we observed that AM attenuated the TNF-*α* and IL-1*β* production in the pleural exudates as well as their expression in the lung tissues from CAR-treated mice. Our results may be supported by earlier *in vivo* studies which show that the peptide reduced the levels of TNF-*α* in bronchoalveolar lavage fluid from rats with LPS-induced acute lung injury [11] and in a model of intestinal ischemia/reperfusion-induced lung injury [20]. In regards to adhesion molecules study, the peptide reduced the upregulation of the expression of ICAM-1 and P-selectin on endothelial cells. Altogether, these data may explain the downregulation of neutrophil infiltration induced by AM treatment in our model of CAR-induced acute lung injury. iNOS-induced NO production has been shown to have proinflammatory action and it has been associated with the initiation and maintenance of different experimental and clinic pathologies including lung diseases; its overproduction leads to oxidative stress that may cause cell death and tissue damage that characterize a number of these pathologies [34, 35]. Our findings demonstrate that AM significantly reduced the iNOS enzyme expression in the lung tissues as well as the production of the stable metabolites of NO in the exudates from CAR-instilled mice. These data are in agreement with previous studies reporting that this peptide inhibited NO production in both experimental colitis [19] and LPS-activated macrophages [17].

Activated neutrophils are reported to be one of the main sources of ROS and RNS, which have been shown to contribute to the pathogenesis of various inflammatory lung injuries and other diseases. Along these lines, they can initiate lipid peroxidation that results in both DNA damage and destruction of the cell membranes involving polyunsaturated fatty acids, which are extremely sensitive to oxidation [36]. In the present study, we indicated that AM treatment prevented the formation of MDA that is considered a good indicator of lipid peroxidation, which reflects reduced oxidative stress. On the other hand, increased production of NO combined with the production of ROS contributes to the formation of highly reactive products such as peroxynitrite. The formation of nitrotyrosine is a marker for the detection of the generation of peroxynitrite and other stress nitrosative derivatives [37]. Our results reveal that the peptide attenuated the increase of nitrotyrosine formation caused by CAR. We propose that this reduction of nitrosative stress by AM may be attributed to the inhibition of iNOS expression, and the subsequent formation of NO. In addition, O_2_^−^, a reactive oxygen species, and peroxynitrite generated during lung injury trigger other cytotoxic effects, including DNA damage. They produce strand breakage of DNA, inducing activation of the nuclear protein PARP. Once activated, it catalyzes the cleavage of nicotinamide adenine dinucleotide (NAD+) into nicotinamide and ADP-ribose and then uses the latter to synthesize polymers of ADP-ribose in DNA repair. However, under conditions of severe DNA injury, overactivation of PARP severely depletes the intracellular stores of NAD+, which affects mitochondrial respiration and cell energetic balance, ultimately leading to cell death by necrosis [38]. In studies of acute lung injury by various causes, PARP was shown to play a pivotal role in the pathogenesis of the injury and PARP inhibitors have therapeutic effects [39–41]. We demonstrate here that AM attenuated the increase in PARP activity caused by CAR instillation. Taken together, these findings report that AM reduced production/expression of markers of nitrosative and oxidative stress and thus DNA damage in lung tissue. Our observations are consistent with previous papers reporting the antioxidant properties of AM. Along these lines, Matsui et al. [42] and Kim et al. [43] have shown a protective effect of AM against hypoxia-induced pulmonary vascular remodelling and hypoxia/reoxygenation-induced cell death, respectively, through the inhibition of ROS production, which might provide an effective therapeutic strategy.

How do adrenomedullin regulate such a wide range of inflammatory and immunomodulatory mediators? The cAMP-PKA pathway is the main intracellular signalling pathway that is involved in most of the effects exerted by all of these neuropeptides, including the downregulation of inflammatory mediators [44]. cAMP-inducing agents have been found to be potent anti-inflammatory factors. They act by downregulating the activation of the nuclear factor-B (NF-B), a factor that is essential for the transcriptional activation of most of the inflammatory cytokines, chemokines and costimulatory factors. These neuropeptides, through increased levels of intracellular cAMP, decrease NF-B nuclear translocation, and DNA binding induced by different bacterial products and cytokines by inhibiting the phosphorylation and subsequent degradation of the inhibitor of NF-*κ*B, IkB, which retains p65 (an essential component of the NF-*κ*B transactivating complex) in the cytoplasm [44].

In this way, Pleguezuelos and Kapas [45] reported that AM was capable of abolishing the NF-*κ*B translocation in stimulated keratinocytes. In accordance with these findings, we demonstrate that AM administration significantly decreased the degradation of I*κ*B-*α* and the nuclear translocation of p65 in lung tissues, 4 h after CAR administration. Combining these results, it can be reported that the reduction of TNF-*α* and IL-1*β* production or expression and iNOS expression by AM described in the present study is most likely attributed to an inhibitory effect in NF-*κ*B activation.

## 5. Conclusions

In summary, our studies led to the conclusion that AM has potential anti-inflammatory actions in the development of CAR-induced pleurisy, attenuating NF-*κ*B activation, and the expression of proinflammatory cytokines and iNOS enzyme. These effects may explain the decrease in neutrophil migration and nitrosative and oxidative stress during the course of the inflammatory response. Taken together, the results of the present study enhance our understanding of the role of the peptide AM in the pathophysiology of the inflammation and support its therapeutic potential for lung inflammatory pathologies.
